# Evidence of Grammatical Knowledge in Apes: An Analysis of Kanzi’s Performance on Reversible Sentences

**DOI:** 10.3389/fpsyg.2022.885605

**Published:** 2022-07-21

**Authors:** P. Thomas Schoenemann

**Affiliations:** Cognitive Science Program and Anthropology Department, Indiana University, Bloomington, IN, United States

**Keywords:** ape, grammar, syntax, evolution, language, argument relations

## Abstract

Ape language acquisition studies have demonstrated that apes can learn arbitrary mappings between different auditory or visual patterns and concepts, satisfying the definition of symbol use. The extent to which apes understand aspects of grammar is less well accepted. On the production side, several studies have shown that apes sometimes combine two or more symbols together, in non-random patterns. However, this is quite limited compared to human language production. On the comprehension side, much greater abilities have been reported in apes. One of the most famous examples is Kanzi, a bonobo who reportedly responded correctly to a large number of novel commands. However, based on his performance on a small subset of reversible sentences—where the understanding of English syntax was critical—the extent to which he demonstrated grammatical knowledge has been questioned. Using a randomization study it is shown here that his performance actually vastly exceeds random chance, supporting the contention that he does in fact understand word order grammatical rules in English. This of course represents only one aspect of English grammar, and does not suggest he has completely human grammatical abilities. However, it does show that he understands one of the arbitrary grammatical devices used in many languages: The use of word order to code argument relations. It also removes from serious consideration the view that apes lack any kind of grammatical ability. From an evolutionary perspective, Kanzi’s ability is most likely to result from homologous brain circuitry, although this is ultimately an empirical question.

## Introduction

Ape language studies have undermined many of the claims of human uniqueness regarding language. Chomsky once claimed “human language appears to be a unique phenomenon, without significant analog in the animal world. If this is so, it is quite senseless to raise the problem of explaining the evolution of human language from more primitive systems of communication that appear at lower levels of intellectual capacity” (Chomsky, 1972, p. 67). Yet the year before this publication Premack (1971) had already reported that the chimpanzee Sarah was able to use physical lexigrams to demonstrate a range of basic features found in human language, including the ability to associate an arbitrary sign with a specific referent, as well as understanding basic aspects of constituent structure coding hierarchical relations among symbols. Subsequent studies have reported a variety of linguistic (or at least language-like) abilities among captive apes, including abilities suggestive of basic grammatical understanding. Nevertheless, the view that these ape language studies have not successfully demonstrated cognitive abilities relevant to human language grammar and language evolution in general can still be found (Wynne, 2008; Berwick et al., 2013). Below a short review of ape language studies particularly relevant to the question of grammatical understanding is given, culminating with one of the most comprehensive assessments of the question to date: Savage-Rumbaugh et al.’s (1993) study of the chimpanzee Kanzi.

## Ape Language Studies

Although early studies attempting to raise chimpanzees as human children suggested some clear understanding of human language (Kellogg and Kellogg, 1933; Hayes and Hayes, 1951), it was clear that producing human speech sounds was very difficult. Subsequent research programs therefore focused on non-vocal linguistic systems. The brief review below focusses on three of these research programs that produced data particularly relevant to the question of whether our closest relatives are able to obtain any grammatical understanding.

Premack (1971) and Premack and Premack (1972) demonstrated that the chimpanzee Sarah had competence on a number of language abilities. Sarah was able to associate different concepts with different arbitrary signs (fulfilling Peirce’s (1867) definition of a symbol). The concepts included not just individual items like “apple” and “banana,” but also broader class concepts like color, shape, and size. A vivid example of her understanding of the meaning of these symbols can be seen in her answer to the question “what color is apple?”: Her lexigram for “apple” was literally colored blue, but her answer to the question was her lexigram for “red.” Furthermore, she demonstrated the ability to understand the hierarchical relations of constituent structure in her language. For example, when asked “Sarah insert apple pail banana dish”—which in her system meant “Sarah put the apple in the pail and the banana in the dish”—she correctly responded, even though “pail” and “banana” were right next to each other in the sequence (suggesting she understood something about the intended hierarchical relationships of the constituents). It is, however, not clear from these reports how many sentences of this type she correctly interpreted, so statistical analysis is not possible.

The Gardner’s work with the chimpanzee Washoe, and later with chimpanzees Moja, Tatu, and Dar, also demonstrated similar symbolic abilities using a sign language rather than lexigrams (Gardner and Gardner, 1994). Double-blind tests showed Washoe learned the meanings of signs and used them correctly. They all also showed clear biases in word order. For example, they were significantly more likely to place terms for persons and objects *after* terms for traits and attributes (Gardner and Gardner, 1994). However, the length of sign strings they produced was generally short, consisting often of only two signs.

The work with the bonobo Kanzi by Savage-Rumbaugh and Rumbaugh (1993) has provided even more evidence of the possibility of linguistic competence in an ape. Early work at the Yerkes Language Research Center by Savage-Rumbaugh and Rumbaugh (1993) focused on training the chimpanzee Lana to differentiate the referents of various lexigrams and to arrange them appropriately to obtain rewards. Work with other chimpanzees later focused on acquisition through a process emphasizing shared intentionality and social interaction (Savage-Rumbaugh and Rumbaugh, 1993). The most impressive success came with their work with the bonobo Kanzi, who was exposed at a very early age to both spoken English and a visual lexigram system, whereby researchers used both when addressing him, but his responses were via lexigrams. He gained proficiency at understanding the meanings of spoken words and their corresponding lexigrams, as demonstrated through double-blind experiments assessing his competency (Savage-Rumbaugh and Rumbaugh, 1993).

In addition, Savage-Rumbaugh et al. (1993) probed Kanzi’s comprehension with 660 spoken English sentences in the form of commands, whereby his responses would allow observers to assess his understanding. 416 of these commands were given “blind,” such that the experimenter giving the command was behind a one-way mirror. Many of these commands were constructed to be novel and unusual (e.g., “put the pine needles in the refrigerator”). The commands used several different kinds of grammatical constructions, but not all of them allow for an assessment specifically of grammatical understanding independent of an understanding of the meanings of individual words. However, a subset of 20 pairs of sentences were particularly interesting with respect to probing grammatical understanding, because each of these pairs were reversible with respect to the order of constituents. An example of such a pair is: “Pour the Coke in the lemonade” and “Pour the lemonade in the Coke.” Correct response to these sentences depends not just on an understanding of individual word meanings, but critically also on an understanding of English syntactic word order rules. The list of these 20 pairs of sentences from Savage-Rumbaugh et al. (1993) are shown in Table 1. He was judged to have correctly responded to 31 of these sentences (78%), partially correct on 7 (18%), and incorrect on only 2 (5%) of them.

**TABLE 1:** Kanzi’s performance on reversible sentences (from Savage-Rumbaugh et al., 1993).

Performance on pair*	Command given	Blind trial? §	Head-phones?¥	Trial number	Original response code**	Savage-Rumbaugh et al. (1993) transcription of Kanzi’s response***
Both correct	Put the ball on the rock.	No	No	48	C	[Transcription not reported]
	Can you put the rock on your ball?	No	No	95	C	[Transcription not reported]

Both correct	Can you put some oil on your ball?	No	No	110	C	[Transcription not reported]
	
	Put the ball in the oil.	Yes	Yes	516	C	(Kanzi does so.)

Both correct	Put some water on the carrot.	No	No	201	C	“Kanzi responded by tossing the carrot outdoors; since it was raining heavily at the time, his action resulted in water getting on the carrot even though he applied the water indirectly. This method of “putting water on the carrot” appeared to be deliberate on Kanzi’s part. At no other time during the test did he toss food or other items outdoors.” (p. 81-82)
	
	Put the carrot in the water.	Yes	Yes	450	C1	(Kanzi picks up a carrot, makes a sound like “carrot,” takes a bite of the carrot, then puts it in the water). [C1 is scored because Kanzi eats some of the carrot before putting it in the water.]

Both correct	Put the pine needles in your ball.	Yes	No	251	C	(Kanzi does so.)
	
	Can you put the ball on the pine needles?	Yes	Yes	588	C	(Kanzi does so.)

Both correct	Pour the Coke in the lemonade.	Yes	Yes	486	C	(Kanzi does so.)
	
	Pour the lemonade in the Coke.	Yes	Yes	488	C	(Kanzi does so, making a sound like “lemonade.”)

Both correct	Put the tomato in the oil.	Yes	Yes	525	C	(Kanzi does so.)
	
	Put some oil in the tomato.	Yes	Yes	528	C	(Kanzi picks up the liquid Baby Magic oil and pours it in a bowl with the tomato.)

Both correct	Pour the juice in the Jello.	Yes	Yes	502	C	(Kanzi does so.)
	
	Open the Jello and pour it in the juice.	Yes	Yes	499	C	(Kanzi opens the Jello and pours it in the juice.)

Both correct	Rose is gonna chase Kanzi.	Yes	Yes	643	C	(Kanzi looks at Rose.) E says, “Rose is going to chase you.” (Kanzi looks at Rose, puts his bowl down, signs chase, points to Rose, then runs away. Rose chases Kanzi.)
	
	Kanzi is going to chase Rose.	Yes	Yes	636	PC	(Kanzi looks at Rose and scoots over toward her, as though waiting for her to run away. Rose does nothing. Kanzi touches Rose. Rose gets up, and Kanzi then backs away, stops, looks at Rose, and waits for her to run. Rose doesn’t, so Kanzi approaches instead.) Error correction. -E tells Rose what is supposed to happen. Kanzi then gestures toward Rose, and she chases him.

Both correct	Liz is going to tickle Kanzi.	Yes	Yes	635	C	(Kanzi looks toward Liz, holds his hand out to her, vocalizes “enngh,” then approaches Liz, then goes over and sits down near her and holds his hand out to her. Liz stands up. Kanzi motions toward himself, then laughs, then signs tickle, then leans down to be tickled. Liz tickles him.)
	
	Kanzi is gonna tickle Liz.	Yes	Yes	655	PC	(Kanzi goes over to Liz and touches her briefly on the leg with his index finger, then backs away. Liz reaches her hand out to him and starts to tickle his neck. He gets down on the floor in a tickle posture.) [Kanzi appeared to be initiating a “tickle” interaction with Liz, but the direction of the interaction was not clear.]

Both correct	Kanzi is going to tickle Liz with the bunny.	Yes	Yes	651	C	(Kanzi picks up the bunny puppet, puts it on his hand, walks over to Liz, and begins tickling her leg. He also tickles Linda.) E says, “Just Liz.” (Kanzi returns to tickling Liz.) E says, “You can come back now.” (Kanzi returns and makes a sound like “ana” as he picks up a piece of banana.)
	
	Liz is going to tickle Kanzi with the bunny.	Yes	Yes	660	PC	(Kanzi stands up and lifts the toy gorilla up briefly. It is laying on top of his keyboard.) E says, “Give Liz the bunny so she can tickle you.” (Kanzi takes the toy gorilla to Liz and drops it on the floor in front of her with a play face. Liz picks it up and begins to tickle Kanzi.)

Both correct	Put the raisins in the water.	Yes	Yes	542	C	(Kanzi takes the raisins out of the round box and puts them in a bowl of water, then makes a sound like “raisin.”)
	
	Pour some water on the raisins.	Yes	Yes	546	C2	(Kanzi picks up the quart of water, holds it for a moment near the bowl of lettuce that he has been eating, and pauses.) E says, “Kanzi, pour some water on the raisins.” (Kanzi does so.)

Both correct	Put the egg in the juice.	Yes	Yes	510	C	(Kanzi makes a sound like “egg,” then does so.) [see also Figure 15 in Savage-Rumbaugh et al. (1993)]
	
	Pour the juice in the egg.	Yes	Yes	507	C3	(Kanzi picks up the bowl with the egg in it, smells it, and shakes the bowl to watch the egg wiggle.) E says, “Pour the juice in the egg.” (Kanzi puts the egg down, picks up the can opener, opens it up, and tries to latch it onto the can.) E says, “It’s already open, just pour it in.” (Kanzi bangs on it with the can opener.) E says, “Kanzi, just pour it. Pick it up and pour it in.” (Kanzi does so.)

Both correct	Pour the Perrier water in the milk.	Yes	Yes	478	C	(Kanzi does so.)
	
	Put the milk in the water.†	Yes	Yes	451 & 456	C	(Kanzi picks up a closed can of SMA [milk], looks at the water, and shakes the milk, trying to figure out how to get the milk out of the can into the water.) E says, “Put the milk,just put the whole can in the water.” (Kanzi looks around for something to open the milk with.) E says, “Just put the can in,just drop the milk in the water. [C is scored because Kanzi’s behavior indicates that he has understood the sentence and is trying to figure out how to open the can so that he can pour the milk in the water. E’s suggestions that he just put the whole can in the water are ignored, probably because, in his experience, the cans of SMA are opened and mixed with water, never just dropped in a bowl of water. Placing a can of milk in a bowl of water seems to make no sense to Kanzi].

Both correct	Make the doggie bite the snake.	Yes	Yes	580	C	(Kanzi picks up the dog and puts it on the snake, then moves it back, picks up the snake, and looks at its mouth.) E says, “Make the doggie bite the snake.” (Kanzi puts the snake’s mouth up to the doggie’s mouth.) E says, “Yeah, that’s right. Un huh. Thank you.” (Kanzi opens the dog’s mouth and sticks the snake’s head in the dog’s mouth.) E says, “Yeah, push his mouth down. Yeah, that’s very good, Kanzi.” (Kanzi pulls the snake back and puts it down.) [C is scored because Kanzi does not hesitate at any point and his actions appear to be directed smoothly toward carrying out the request.]
	
	Make the snake bite the doggie.	Yes	Yes	585	C	(Kanzi picks up the snake and then the dog. Kanzi pushes the snake’s mouth down onto the dog’s mouth.) E says, “Uh huh, that’s real good.” (Kanzi holds the snake’s mouth on the doggie’s mouth.) (p. 193)
Correct-incorrect	Kanzi is going to chase Liz.	Yes	Yes	648	C	(Kanzi looks around but stays seated.) E says, “Kanzi is going to chase Liz.” (Kanzi goes over to Liz, taps her on the leg, and moves away. She gets up and chases him.)
	
	Liz is gonna chase Kanzi.	Yes	Yes	631	C	(Kanzi interrupts.).*Go tell her, go tell her to chase you*. (Kanzi goes over to Liz, touches her, and moves away. Liz interprets this as a request to chase and chases Kanzi.)

Correct-incorrect	Put the rock in the water.	Yes	Yes	444	C	(Kanzi does so.)
	
	Pour the water on the rock. Pour the water.	Yes	Yes	447	PC	(Kanzi takes the rock out of the bowl and puts it in the bin, which is filled with water.) E says, “Pour the water out.” (Kanzi puts the bowl in the bin of water with the rock and gets some water in the bowl.) Error correction.-E says, “Can you pick it up? Yeah, that’s right, pour some of it out on the rock.” (Kanzi lifts the bowl full of water to his mouth, takes a sip, and sets it down on the floor.)

Correct-incorrect	Pour the cereal in the milk.	Yes	Yes	557	C	(Kanzi picks up a box of cereal, holds it with his foot, pulls the spout out, and pours it in the milk.)
	
	Pour the milk in the cereal.	Yes	Yes	552	PC	(Kanzi picks up the cereal and opens the box, then pours the cereal into a bowl of other cereal, then into a bowl of milk.) Error correction. -E says, “hat’s good, put it down.” (Kanzi pours the cereal into a bowl of other cereal again.) E says, “Stop.” (Kanzi pours it into the bowl of milk.) E says, “Put the box down.” (Kanzi continues to pour.) E says, “That’s enough.” (Kanzi continues to pour.) E says, “Stop.” (Kanzi continues to pour.)

Correct-incorrect	Put the raisins in the shoe.	Yes	Yes	543	C2	(Kanzi places his hand on the quart water jar and pauses.) E says, “Put the raisins in the shoe.” (Kanzi touches the melon and the shoe.) E says, “That’s good, Kanzi, put some raisins in the shoe. Uh huh.” (Kanzi takes some raisins out of the water and puts them in the shoe.)
	
	Put the shoe in the raisins.	Yes	Yes	634	I	(Kanzi picks up the raisins, opens them up, and puts them in a bowl.) E says, “OK, now put the shoe in the raisins. (Kanzi puts one tiny raisin in the shoe, then proceeds to untie the shoe.) [I is scored instead of PC because taking the raisins out of the box and putting them in a bowl is something that is often done with the raisins prior to acting on them in some other manner and probably does not reflect Kanzi’s attempt to respond to the sentence.]

Correct-incorrect	Put the tomatoes in the melon.	Yes	Yes	469	C3	(Kanzi looks around and appears hesitant and puzzled.) E says, “Put the tomato in the melon.” (Kanzi picks up a little tomato and puts it in the melon.) [C3 is scored because the minor rephrasing here, from the plural to the singular, may have helped Kanzi, who seemed immediately to know what to do after the sentence was rephrased. The rephrasing from plural to singular was intentional and was given in response to the puzzled expression on Kanzi’s face.]
	
	Put the melon in the tomatoes.	Yes	Yes	465	PC	(Kanzi puts the melon in the water.) Error correction. -E says, “Put the melon in the tomato.” (Kanzi takes the melon out and puts it on top of a quart bottle that has water in it.) E says, “In the tomatoes.” (Kanzi puts the melon in the tomatoes.)

Both incorrect	Put the hat on your ball.	Yes	Yes	569	PC	(Kanzi picks up the shoe and plays with it next to the ball.) E says, “Put the hat on your ball.” (Kanzi continues to play with the shoe.) E says, “The hat, not the shoe.” (Kanzi says, “Whuuh,” and continues to play with the shoe, trying to take the laces out of it.) E says, “Put the hat, do you see the hat?” (Kanzi continues to play with the shoe and does not even look for the hat.) Error correction. -E says, “You don’t see the hat?” (Kanzi continues to play with the shoe.) E says, “Look for the hat.” (Kanzi points to the hat.) E says, “That’s the hat. Put the hat on your ball.” (Kanzi puts the hat on the ball.)
	
	Put the ball on the hat.	Yes	Yes	599	I	(Loud screaming from Tamuli [another bonobo] drowns out the sentence.) … Put the ball, put the ball on the hat. (Kanzi picks up the hat and puts it on the ball.)

*Color coding:*

*Orange 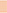 : Trial was not blind (see column 3), or other individuals in the room were not wearing headphones playing loud music (see column 4), or both.*

*Yellow 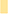 : Trials for which Wynne finds the coding “extremely” (2006) or “unreasonably” (2008) generous.*

*Light gray 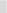 : Sentence pairs where one response was treated by the analysis here as correct, but the other incorrect.*

*Dark gray 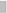 : Sentence pairs where both responses were treated by the analysis here as incorrect.*

*^†^ “Put the milk in the water” was administered twice, once with the milk can open, instead of closed. It was counted only once for purposes of the analysis.*

* As interpreted for the analysis given in the text. Note that in most cases sentences coded as Partial Correct (PC) by Savage-Rumbaugh et al. (1993) were here interpreted as incorrect with respect to Kanzi’s understanding of the grammar.

*§ Was experimenter giving the command behind a one-way mirror?*

*¥ Were the other individuals in the room wearing headphones playing loud music (so they couldn’t hear the command)?*

** Original response codes:

*C, Kanzi carries out the request immediately and correctly; C1, Kanzi first hesitates or engages in a tangential activity, then carries out the request correctly; C2, Kanzi first hesitates or engages in a tangential activity, the Experimenter repeats the request, then Kanzi carries out the request promptly; C3, Kanzi first hesitates or engages in a tangential activity, the Experimenter rewords and may repeat the request, then Kanzi carries out the request promptly; PC, Kanzi is partially correct in carrying out the request; I, Kanzi carries out the request in inverse order but is correct with respect to all other components of the request.*

*** from Savage-Rumbaugh et al. (1993).

Savage-Rumbaugh et al. (2009) note that there were a number of additional sentences for which word order was also inverted, and that Kanzi was correct on 81% of these. However, they are not as clean examples with respect to probing syntactic understanding, because they include semantic clues from individual words that could arguably bias Kanzi’s behavior toward correct responses even if he lacked understanding of the underlying syntax. An example of these additional pairs of sentences are: “Take the snake outdoors” vs. “Go get the snake that’s outdoors.” The concern is that Kanzi might be able to respond correctly (above chance) to these sentences if he simply understood the difference in meanings of “take” and “go get” (in addition to “snake” and “outdoors”), rather than actually understanding the underlying English syntax. Note here that such a critique rests on the assumption that Kanzi *does in fact understand individual words*—which is a fundamental component of language and implies this basic ability predated human evolution. The focus of recent critiques of ape language studies, however, has been on the question of grammar.

## Critique of Kanzi’s Performance on Reversible Sentences

Wynne argues that Savage-Rumbaugh et al. (1993) were either “extremely generous” (2006, p. 125) or “unreasonably generous” (2008, p. 14) in their determination of whether Kanzi’s responses indicated understanding. He admits to being “nitpicky,” but argues that this is “to see if he understands grammar” (Wynne, 2008, p. 14), and claims “…this test is the only evidence we have for whether or not any non-human comprehends grammatical utterances” (Wynne, 2006, p. 125). He gives several examples that he thinks clearly show unreasonable generosity, and concludes that “…on any assessment not tinted with rose-colored glasses, Kanzi just doesn’t get it” (Wynne, 2008, p. 14). In their response to Wynne’s (2008) critique, Savage-Rumbaugh et al. (2009) note that the majority of Kanzi’s errors on these reversible sentences were *not* errors of inversion—which would indicate true errors of understanding the syntax (see also Savage-Rumbaugh and Rumbaugh, 1993).

An examination of examples Wynne gives of either “extremely” (2006) or “unreasonably” (2008) generous coding are shown below, with Wynne’s own (2008) statements on why he finds the coding by Savage-Rumbaugh et al. (1993) too generous (note that Wynne, 2006, contains almost identical wording). This is followed by the actual reported response detailed in Savage-Rumbaugh et al. (1993), and an assessment of Wynne’s claim given this. Note again that the critical issue is whether Kanzi understands grammar—the *nature* of any errors is therefore fundamentally important.

(1) Wynne (2008, p. 14) complains that when Kanzi was asked to “Pour the juice in the egg” “Kanzi proceeded to pick up the bowl with the egg in it, sniff it, and shake it. They repeated the command three times—each time changing the wording slightly—before Kanzi did what they asked him to. They nonetheless scored his response as correct.”

*Actual Reported Response (Table 1):*
“(Kanzi picks up the bowl with the egg in it, smells it, and shakes the bowl to watch the egg wiggle).” E says, “Pour the juice in the egg.” (Kanzi puts the egg down, picks up the can opener, opens it up, and tries to latch it onto the can). E says, “It’s already open, just pour it in.” (Kanzi bangs on it with the can opener). E says, “Kanzi, just pour it. Pick it up and pour it in.” (Kanzi does so).
*Assessment*: Wynne’s summary of the actual reported response is clearly inaccurate in a manner that is also misleading. If Kanzi really didn’t understand the syntax, he would have proceeded to pour the egg in the juice, yet he did not do so. After having the command repeated the first time, he still does not pour the egg in the juice, but instead picks up the device one would need to open the juice can to allow it to be poured. The rest of his actions are consistent with his understanding the syntax.
*Conclusion*: Kanzi’s responses Are consistent With his understanding the syntax of these sentences.

(2) Wynne (2008, p. 14) states: “When they asked Kanzi to ‘Pour some water on the raisins,’ he held a jug of water over a lettuce. This was coded as correct.”

*Actual Reported Response (Table 1):*
“(Kanzi picks up the quart of water, holds it for a moment near the bowl of lettuce that he has been eating, and pauses). E says, ‘Kanzi, pour some water on the raisins.’ (Kanzi does so).”
*Assessment*: Wynne fails to mention the critical context that Kanzi had been eating lettuce from the bowl in question, as well as that Kanzi did not pour any water in the lettuce. If Kanzi really had no idea about English syntax, he should have been equally likely to “pour the raisins on the water” as to “pour the water on the raisins.” Furthermore, even if he had poured the water into the lettuce bowl this would not count as an incorrect interpretation of English syntax, but instead would have been an incorrect interpretation of the meaning of the word “raisins” (mistaking it for “lettuce”).
*Conclusion*: The errors are not relevant to the question of syntax. Kanzi’s responses are consistent with his understanding the syntax of these sentences.

(3) Wynne (2008, p. 14) states: “Kanzi’s first reaction to the request to pour milk into water was to stick a tomato in the water.”

*Actual Reported Response (Table 1):*
“(Kanzi picks up a closed can of SMA [milk], looks at the water, and shakes the milk, trying to figure out how to get the milk out of the can into the water.) E says, “Put the milk, just put the whole can in the water.” (Kanzi looks around for something to open the milk with). E says, ‘Just put the can in, just drop the milk in the water.’ [C is scored because Kanzi’s behavior indicates that he has understood the sentence and is trying to figure out how to open the can so that he can pour the milk in the water. E’s suggestions that he just put the whole can in the water are ignored, probably because, in his experience, the cans of SMA are opened and mixed with water, never just dropped in a bowl of water. Placing a can of milk in a bowl of water seems to make no sense to Kanzi.”
After this episode, the same command was given to Kanzi again (5 separate commands later). For this second trial on this command, his reported response was:
“(Kanzi is still poking the tomato with his thumb from [the previous] trial 455 while he listens to the sentence. After the sentence, he picks up that tomato and puts it in the water.) E says, “Put the milk in the water.” (Kanzi pours the milk in the water)” They go on to note: “[This is a re-presentation of trial 451 [the first time Kanzi was asked given this command] to determine what Kanzi will do if the container of milk is open, rather than closed, when the sentence is presented. The fact that Kanzi now pours the milk directly into the water validates the interpretation of the difficulty he encountered on trial 451].”
*Assessment*: Wynne neglects to mention that Kanzi’s error actually occurred on the *second* trial of the same sentence. On first trial Kanzi appears to think he needed to open the can of milk first before pouring it. This is not an error of syntax. The error Wynne highlights on the second trial is not one of syntax either, but—if anything—involved misunderstanding the meaning of a single word. Hence, Wynne’s summary is highly misleading and irrelevant.
*Conclusion*: The errors are not relevant to the question of syntax. Kanzi’s responses are consistent with his understanding the syntax of these sentences.

(4) Wynne (2008, p. 14) states: “When asked to chase Liz he remained seated; when asked again he touched Liz’s leg and she chased him.”

*Actual Reported Response (Table 1):*
“(Kanzi looks around but stays seated). E says, “Kanzi is going to chase Liz.” (Kanzi goes over to Liz, taps her on the leg, and moves away. She gets up and chases him).”
*Assessment*: Wynne neglects to mention that the actual command was: “Kanzi is going to chase Liz” and not: “Kanzi, chase Liz.” These have different implications for English speakers, so an initial initial lack of response by Kanzi might be expected. When repeated, he demonstrates he knows Liz is involved, though it is unclear what he thinks has been asked with respect to who is supposed to chase whom. Since he had always been expected to defer to his human caretakers, we can reasonably assume that when Liz suddenly chases him (which she should not have done, given the command given to him) he would have immediately adjusted his behavior in response to this. However, it is true that this is not a clear indication of his understanding of syntax—though it would be equally wrong to code this as “incorrect” given these circumstances. It is also notable that he was given the same command pair with another third party (“Rose is gonna chase Kanzi” and “Kanzi is going to chase Rose”) and responded correctly to both of those.
*Conclusion*: It is not clear what Kanzi understood about this command from the syntax.

(5) Wynne (2008, p. 14) states: “When Kanzi was given the two commands, ‘Make the (toy) doggie bite the (toy) snake’ and ‘Make the snake bite the doggie,’ in both cases the snake ended up in the dog’s mouth but both responses were coded as correct.”

*Actual Reported Responses* (Table 1):
For “Make the doggie bite the snake”:
“(Kanzi picks up the dog and puts it on the snake, then moves it back, picks up the snake, and looks at its mouth). E says, “Make the doggie bite the snake.” (Kanzi puts the snake’s mouth up to the doggie’s mouth). E says, “Yeah, that’s right. Un huh. Thank you.” (Kanzi opens the dog’s mouth and sticks the snake’s head in the dog’s mouth). E says, “Yeah, push his mouth down. Yeah, that’s very good, Kanzi.” (Kanzi pulls the snake back and puts it down). [C is scored because Kanzi does not hesitate at any point” (p. 192).
For “Make the snake bite the doggie”:
(Kanzi picks up the snake and then the dog. Kanzi pushes the snake’s mouth down onto the dog’s mouth). E says, “Uh huh, that’s real good.” (Kanzi holds the snake’s mouth on the doggie’s mouth) (p. 193).
They also note: “In both instances, Kanzi picked up the agent first and moved the agent toward the recipient” (p. 101). In addition, images are provided of Kanzi’s actions for “Make the doggie bite the snake” (their fig. 14), which appear to show this.
*Assessment*: For the first command, the snake *should* end up in the doggie’s mouth if Kanzi actually understands the syntax, so it is unclear what Wynne’s issue is (a rhetorical technique to confuse the issue?). For the second command, Wynne incorrectly claims that the “the snake ended up in the dog’s mouth,” when in the actual report states that the snake was pushed “onto the dog’s mouth.” Wynne also fails to mention that Kanzi’s actions matched what would be expected if he understood who was supposed to bite whom.
*Conclusion*: Kanzi’s responses are consistent with his understanding the syntax of these sentences.

In summary, only one of the actual examples Wynne raises (i.e., “Kanzi is going to chase Liz”) appears to hold up as an actual case of either “extremely…” (2006) or “unreasonably” (2008) generous coding of Kanzi’s responses with respect to his understanding of the syntax, and even that is balanced by Kanzi’s correct response on the same kind of command given with another agent (“Kanzi is going to chase Rose,” as well as its inverse: “Rose is gonna chase Kanzi”).

### Statistical Analysis

Reasonable people can of course disagree about interpretations. However, neither of Wynne’s (2006, 2008) discussions of Kanzi’s performance includes any attempt at a principled statistical analysis. Wynne (2008, p. 14) states that Kanzi only got a “modest 57% correct” of the reversible sentences correct if we take Savage-Rumbaugh et al.’s (1993) coding of his responses at face value, but less than 30% correct if we don’t. He does not address the statistical significance of these numbers, and instead relies on the intuition that 57% is only a bit over guessing. While it is true that—for any given sentence of this type (i.e., where only an understanding of syntax will allow Kanzi to react appropriately with respect to argument structure)—Kanzi would be expected to get 50% of them right simply by chance. Take for example the command: “Pour the lemonade in the Coke.” If Kanzi only knows the meanings of individual words, but doesn’t understand word order syntax in English, he will be just as likely to pour lemonade into coke (the correct response) as to pour coke into lemonade (the incorrect response).

However, these sentences were paired with reversed versions. Thus, for each pair there are actually four possible outcomes by random chance: (1) He gets both of them wrong, (2) he gets the first of a pair right but the other wrong, (3) he gets the first of a pair wrong but the other right, and (4) he gets both right. Each of these are equally likely, and thus each outcome has a 25% likelihood purely by chance (Table 2). Because the order of each pair of sentences is arbitrary, we can collapse outcomes 2 and 3 into one, with a 50% likelihood (i.e., of one right and one wrong).

**TABLE 2:** Likelihoods of different combinations of random chance responses for pairs of sentences.

		“Pour the coke in the lemonade”*	
		Correct	Incorrect
“Pour the lemonade in the Coke”*	**Correct**	25%	25%
	**Incorrect**	25%	25%

*As examples.

What then was Kanzi’s actual performance? If we take the 20 pairs of sentences that are truly reversible and analyze Savage-Rumbaugh et al.’s (1993) coding and transcriptions of Kanzi’s responses, we can identify which sentences he responded to in a manner consistent with the grammar. Savage-Rumbaugh and Rumbaugh (1993) and Savage-Rumbaugh et al. (2009) point out that most of Kanzi’s errors on these sentences were not errors of inversion (e.g., pouring Coke in lemonade when the reverse was asked), and therefore were often not evidence of a lack of understanding of the syntax of the sentences. Table 1 lists the pairs of reversible sentences, along with the researchers’ transcriptions, an assessment (by this author) of whether the responses were both correct, one correct and one incorrect, or both wrong with respect to syntactic understanding for all 20 pairs of sentences (first column). This accounting indicates Kanzi performed appropriately given the syntax in 14 of those pairs (70%—not 57%), in another 5 pairs he got one right and the other wrong, and in only 1 pair he got both wrong. It is evident that his performance is not close to the random expectations (Table 3).

**TABLE 3:** Comparison of random guessing expected performance vs. Kanzi’s actual performance for pairs of sentences.

	Guessing expected #	Kanzi’s performance*	Guessing expected%	Kanzi%
**Both correct**	5	14	25%	70%
**One correct and the other incorrect**	10	5	50%	25%
**Both incorrect**	5	1	25%	5%

*As coded in the current analysis with respect to consistency with the grammar.

How unusual is this performance, assuming he truly “doesn’t get it” and is simply guessing (“flipping a coin”) with respect to the argument structure coded by syntax? To assess the statistical likelihood of his performance with respect to chance, a simple random simulation study was carried out. This simulation took advantage of the fact that—with respect to understanding the syntax of these reversible sentences—there are only two relevant behavioral responses possible, with one being correct and the other incorrect. The simulation proceeded by (virtually) flipping a coin twice, 20 times in a row, and tabulating the number of times these pairs of flips resulted in either two “heads” (representing the case where both responses are consistent with the syntax), one “head” and one “tail” (representing an instance where one is consistent with the syntax and the other is not), and two “tails” (representing the case where neither responses are consistent with the syntax). Instead of actually flipping coins, either a 0 or 1 is randomly chosen each iteration, with 0 representing an incorrect response and 1 representing a correct response. If the flips are truly random, then given enough iterations of pairs of flips, the proportions of each possible outcome in Table 2 will approach 25%. To determine the probability of matching Kanzi’s actual performance (14 pairs both correct, 5 pairs only one is correct, and 1 pair both were wrong), we iterate this process of 20 pairs of virtual coin flips a large number of times—in this case we repeated the process 1,000,000 times—and tabulate the results (see Appendix for the code used to do this in the R Statistical Package). It turns out that the odds of getting at least 14 pairs both correct combined with at least 5 pairs where only 1 is correct—completely by chance—is *p* < 0.00001.

This analysis of Kanzi’s performance does assume that some sort of inadvertent cuing (“Clever Hans effect”) did not significantly bias his responses toward correct performance on the commands analyzed here. While this cannot be absolutely ruled out, the experimenters did go to great lengths to minimize the possibility. For the first 244 trials the person giving the command sat in front of Kanzi (Savage-Rumbaugh et al., 1993, p. 50). However, trials 245–660 were blind, where the command was given from behind a one-way mirror. They do note that this situation was apparently strange to Kanzi, and that if he was hesitant or incorrect “the experimenter came out from behind the screen to help carry out a sentence” (Savage-Rumbaugh et al., 1993, p. 51). However, hesitancy and incorrectness is noted in their transcriptions (i.e., there is no indication that Kanzi was scored as correct on such trials; Table 1). However, most of the reversible sentences that are the focus of the present analysis were in fact presented blind in this way (a total of only 4 out of 40 sentences). If we count only the 17 paired sentences for which both had been presented blind (i.e., excluding the first 3 pairs in Table 1; see column 3), Kanzi’s performance was still 11 pairs both correct, 5 pairs only one is correct, and 1 pair both wrong. The odds of this happening are only *p* < 0.00018.

For some of the sentences Kanzi was expected to interact with other experimenters. Could they have inadvertently cued him? These individuals were located 3–10 feet from Kanzi, and “…were generally located behind him” (Savage-Rumbaugh et al., 1993, p. 47). For the first 100 blind trials (trials 245–344) only, these individuals were instructed to respond appropriately “…when the action was specifically directed to them… Note, however, that these individuals were located well away from the subject and spaced far apart; additionally, they kept their eyes covered until the subject had selected the correct object and approached them” (Savage-Rumbaugh et al., 1993, p. 51). According to the researchers Kanzi did not typically look for cues, and “…failure to carry out the request appropriately did not prompt (Kanzi) to do so either” (Savage-Rumbaugh et al., 1993, p. 52). After trial 344, these individuals wore headphones playing loud music, thereby preventing them from hearing the commands given Kanzi. However, only 5 of the 40 sentences at issue here were presented with the other individuals not wearing headphones, which accounts for only one additional paired sentence group. Excluding that from the analysis leaves Kanzi’s performance at 10 pairs both correct, 5 pairs only one is correct, and 1 pair both wrong. The odds of this happening if Kanzi was only guessing is only *p* < 0.0006.

What if we take Wynne’s (2008) “nitpicky” assessments at face value, even though they appear to be highly misleading and flawed, as outlined above? Counting all 20 pairs, Kanzi would be coded as having gotten both correct on 10 pairs, only one correct on 9 pairs, and both wrong on only 1 pair. The odds of this happening by chance (i.e., he was simply guessing about syntax) are still only *p* < 0.0011. If we additionally exclude the pairs for which at least one of the pair was not given with the researcher behind a one-way mirror, leaving 17 pairs in total, Kanzi still got 7 of these both correct, 9 where only one was correct, and 1 where both were wrong, which would have a probability of only *p* < 0.008 if he really was just guessing about the syntax. Even if we also exclude the (additional) sentence pair for which other individuals in the room were not wearing headphones to mask them from hearing the commands, Kanzi still got 6 pairs both correct, 9 pairs only one is correct, and 1 pair both wrong, which would have a probability of only *p* < 0.013 if he was just guessing about the syntax.

This shows that Wynne’s (2006, 2008) intuition about the likelihood of Kanzi’s performance with respect to syntax is actually incorrect. Kanzi’s performance is vastly better than chance expectations, indicating that he was indeed sensitive to the syntactic cues. Kanzi apparently *does* “get it,” even without rose-colored glasses.

## Discussion

The pairs of reversible sentences analyzed here from Savage-Rumbaugh et al. (1993) are particularly useful for probing the understanding of an arbitrary symbolic grammatical rule system that is fundamental to English and many other languages. This is because simply understanding individual word meanings is not sufficient to allow above chance performance with respect to argument structure in response to these commands (Wynne, 2006, 2008). Responding above chance depends on understanding the word order rules. Furthermore, the argument relations that are coded by these rules are hierarchical: one constituent lies at a different level of the thematic hierarchy than the other. For example, in the command “Pour the Coke in the lemonade” the syntax demands that the two nouns differ with respect to which is to be actively manipulated (“Coke”) and which is to passively accept the other (“lemonade”). Similarly, the syntax of the command “Kanzi is going to chase Rose” demands that Kanzi is to pursue Rose, not the other way around. So although the syntactic rule itself uses sequential word order (which is just how English codes argument structure), the coded meaning itself is hierarchical. The analysis presented here thus provides strong support for the contention that at least one ape can learn arbitrary rules coding hierarchical argument relations. Claims that the evidence is weak or non-existent (Wynne, 2006, 2008; Bolhuis et al., 2014) are therefore incorrect.

There are limitations of the present analysis that should be noted. First, it of course depends on the faithfulness of the transcriptions of Kanzi’s responses as documented in Savage-Rumbaugh et al. (1993). These have not to my knowledge ever been seriously questioned. All but 60 of 660 total trials were video recorded (failures being ascribed to camera malfunction or Kanzi moving out of view) (Savage-Rumbaugh et al., 1993, p. 67). Transcriptions of these video recordings were made by a separate researcher, after which both Savage-Rumbaugh and a third researcher independently assigned response codes (Table 1, column 6) based on these transcriptions. The Cohen’s kappa on their agreement regarding these codes was 0.72 (Savage-Rumbaugh et al., 1993, p. 72). They do note that “… although different observers coded the data, it was nonetheless the case that the real-time decisions made by SSR … affected all subsequent data coding” in the sense that “real-time interpretations of the subjects’ reactions determined how soon and what type of help was given when the subjects did not carry out a request fairly quickly after it was made” (Savage-Rumbaugh et al., 1993, p. 73).

With respect to the possibility of inadvertently cuing, as noted above Kanzi was unable to see the experimenter giving the command (who was behind a one-way mirror) for most of the trials (all but 4 of the 40 sentences at issue here). In addition, the other individuals in the room with whom Kanzi might be asked to interact with were wearing headphones playing loud music (all but 5 of the 40 sentences) (Table 1, columns 3, 4). Removing commands lacking these safeguards does not change the basic conclusions of the statistical analysis presented here.

Lastly, this was of course the performance of only one bonobo, and how representative he might be for his species is not easy to answer (to the extent this is taken to be a true limitation, more funding should obviously be given to research in this area). However, if one bonobo can learn English word order syntax, it by definition lies within the possibility of extant bonobos, and was also likely true of the common ancestor we (and chimpanzees) share with them.

It is true that these particular sentences only probe one feature of English grammar—not the totality of grammatical features that a fluent English speaker is typically sensitive to. Truswell (2017) has, for example, argued Kanzi does not demonstrate unequivocal understanding of a particular type of noun phrase coordination: where he is expected to apply the same action to two different objects (e.g., ‘Fetch the ball and the rock’). Human language grammar is more than just word order, and some languages (e.g., Latin) do not mark argument structure with word order rules at all. However, it is also true that word order is critical even in Latin: Devine and Stephens (2006) note that it “is not a subject anyone reading Latin can afford to ignore” (2006, p. 6) because it carries all sorts of information about intended meaning. The general ability to decode information from temporal order cues is in fact fundamental to all languages. As such, the ability of an ape to understand how it is used to code meaning is therefore of fundamental importance to any model of how language evolved.

Does Kanzi’s ability with these reversible sentences suggest anything about recursion? The use of recursion by human language grammar has been hypothesized to be a uniquely evolved, language-specific ability (Hauser et al., 2002; Pinker and Jackendoff, 2005; Bolhuis et al., 2014). Such suggestions are undermined by the claim that recursion is not universal across languages (Everett, 2005; Evans and Levinson, 2009). Evolutionary models that posit natural selection for cognitive abilities specific to language, but for which those cognitive abilities are then (after having evolved) not universally used in languages, are highly problematic and likely incorrect (Schoenemann, 1999). Nevertheless, baboons have been shown to display recursive, center-embedded structure in their responses to a memory task (Rey et al., 2012). Furthermore, by at least one reading of what would constitute evidence of recursion (Vicari and Adenzato, 2014), Kanzi’s ability to understand the argument structure of reversible English commands would show that he does have the ability to understand simple recursion. If this is indeed evidence of recursion, it suggests that incipient recursion abilities are not language- or even human-specific, having apparently predated the human lineage.

Because evolutionary change usually occurs through the modification of pre-existing features (anatomical or behavioral), and not the wholesale creation of completely new features (Bock, 1959; Mayr, 1960; Jacob, 1977; Dawkins, 1982), the default evolutionary expectation is that homologies are the rule, not the exception, for language just as with other cognitive abilities (Schoenemann, 1999, 2012, 2018). Therefore, if we find some common cognitive ability in humans and apes—in this case, the ability to use word order to mark hierarchical argument relations—our starting assumption should be that this is due to homologous circuits, not analogous ones. This is of course ultimately an empirical question, regardless of evolutionary theoretical predictions, but from an evolutionary perspective it is simply not the case that domain-specificity and domain-generality are equally likely explanations *a priori*.

Kanzi’s performance with these reversible sentences—building on prior work at the Yerkes with Lana, Sherman, Austin, and others (Savage-Rumbaugh et al., 1993; Savage-Rumbaugh and Rumbaugh, 1993), as well as Sarah (Premack, 1971; Premack and Premack, 1972) and Washoe, Moja, Tatu, and Dar (Gardner and Gardner, 1994)—suggests apes can learn to understand an arbitrary symbolic system to code argument structure. The fact that Nim Chimsky (Terrace et al., 1979) apparently did not show these abilities is of course not relevant to whether these other ape research programs using different methods *were* successful (there are many reasons why a research methodology may fail to show some cognitive ability in an animal *besides* that the animal actually lacks that ability, e.g., inadequate motivation, inadequate learning protocol, etc. (Essock-Vitale and Seyfarth, 1986)). The claim that since human language abilities are much richer than those found among non-human animals, this fact by itself rules out any meaningful continuity (Berwick et al., 2013) is similarly misguided. A difference in richness is good evidence for a difference in degree, it is not good evidence for a difference in kind. A recognition of the basic language-relevant cognitive abilities in apes and other non-human animals, regardless of how limited it might be in these species, is foundational for understanding of how language evolved.

## Data Availability Statement

The raw data supporting the conclusions of this article were taken from Savage-Rumbaugh et al. (1993).

## Ethics Statement

Ethical review and approval was not required for the animal study because it exclusively uses data already previously published by other researchers.

## Author Contributions

PTS conceived the study, carried out the analysis, and wrote the manuscript.

## Conflict of Interest

The author declares that the research was conducted in the absence of any commercial or financial relationships that could be construed as a potential conflict of interest.

## Publisher’s Note

All claims expressed in this article are solely those of the authors and do not necessarily represent those of their affiliated organizations, or those of the publisher, the editors and the reviewers. Any product that may be evaluated in this article, or claim that may be made by its manufacturer, is not guaranteed or endorsed by the publisher.

## Appendix

The code for running the randomization study discussed in the main text using the R Statistical Package (R Core Team, 2021) is:
